# Editorial: Towards continued and affordable accessibility of innovative drugs: sustainable development and efficient use of medicines

**DOI:** 10.3389/fphar.2024.1490853

**Published:** 2024-11-05

**Authors:** Atse H. Huisman, Denise van den Berg, Saco de Visser, Bettina Ryll, Sahar Barjesteh van Waalwijk van Doorn-Khosrovani

**Affiliations:** ^1^ Department of Medical Oncology, Leiden University Medical Centre, Leiden, Netherlands; ^2^ Department of Health Policy, Health Insurers Netherlands, Zeist, Netherlands; ^3^ Treatmeds Foundation, 's-Hertogenbosch, Netherlands; ^4^ Centre for Future Affordable and Sustainable Therapy Development (FAST), The Hague, Netherlands; ^5^ Melanoma Patient Network Europe, Uppsala, Sweden; ^6^ Stockholm School of Economics Institute for Research, Stockholm, Sweden; ^7^ Medical Department, CZ Health Insurance, Tilburg, Netherlands

**Keywords:** affordability and accessibility of medicines, expensive drugs, access, compounding, de-escalation, cost-efectiveness, repurposing, precision medicine

The rising cost of medicines burdens healthcare systems and limits access to novel therapies worldwide. Therefore, sustainable solutions to enhance access and foster innovation are crucial. To highlight current strategies and exchange ideas, we created a Research Topic titled “Towards continued and affordable accessibility of innovative drugs: sustainable development and efficient use of medicines.” Our primary goal was to provide practical recommendations and insights to support healthcare systems. Here we discuss the key topics covered in the Research Topic.

## 1 Addressing uncertainty regarding clinical value

Health Technology Assessment (HTA) bodies and healthcare funders often evaluate the clinical value of new drugs before reimbursement. Several factors can contribute to uncertainty regarding the clinical value; e.g., the validity of surrogate endpoints, concerns about generalisability and lack of long-term efficacy data. In our Research Topic, (Vallano et al.) emphasize the importance of evaluating clinically relevant variables (i.e., overall survival and quality of life) over surrogate endpoints. Broader eligibility criteria can improve the real-world representativeness of clinical studies.

Fagereng et al. studied the impact of these uncertainties on reimbursement decisions in Norway. Drugs with higher certainty of relative effectiveness were more likely to be reimbursed, and at higher costs, than those with lower certainty. This underscores the importance of robust relative-effectiveness data for guiding policy and resource allocation.

In the Netherlands, rising healthcare costs have resulted in a halt in automatic reimbursement for new drugs with a high budget impact used in hospitals. Since 2015, a so-called 'Coverage Lock’ has been implemented by the government to assess these drugs and establish financial arrangements with the manufacturers. Bomhof et al. explored the ethical aspects of reduced drug access under this policy. Although most stakeholders interviewed favoured access through free-of-charge programmes by manufacturers during the Coverage Lock, they expressed concerns about the lack of transparency and unequal access. Creating a national platform such as the Dutch Drug-Access Protocol (Zeverijn et al., 2022), that provides equal access, gathers real-world data, and incorporates a pragmatic, outcome-based risk-sharing model, as well as finding common ground with pharmaceutical companies (Dane et al.), may offer a solution.

Another common challenge discussed in our Research Topic was access to Advanced Therapy Medicinal Products (ATMPs). Rejon-Parrilla et al. identified barriers, such as high initial costs and insufficient long-term effectiveness data, which can burden healthcare systems. With the new HTA regulation starting in 2025, anticancer drugs and ATMPs will undergo joint clinical assessments. This could enable collaborative evidence generation and potentially improve access in the future.

## 2 Increase treatment (cost)effectiveness by preventing overtreatment and de-escalation strategies

With the launch of every new therapy, treatment optimisation studies are essential for refining drug use, improving patient outcomes, and, when possible, enhancing cost-effectiveness. Subjecting patients to unnecessary long treatment duration or high doses of medicine exposes them to avoidable side effects, which can negatively impact their quality of life. Furthermore, overtreatment strains the environment and healthcare resources, including time, personnel and facilities. Pharmaceutical companies themselves usually lack incentives to address overtreatment or explore alternative dosing regimens, as this can slow down or jeopardise developmental or business outcomes. Once the drug is commercially available, a personalised approach (Walia and Prasad) and exploring de-escalation strategies (Buma et al.; van Riel et al.; Dane et al.) are in the interest of both patients, payers, healthcare professionals and society. In our Research Topic, Walia and Prasad challenge the use of an indefinite anticoagulation strategy for patients with unprovoked venous thromboembolism and Buma et al. explore de-escalation regimens of immune-checkpoint inhibitors in lung cancer, alongside extensive biomarker research to address overtreatment (van Riel et al.). The latter is funded by a national fund (Treatmeds Foundation) (Barjesteh van Waalwijk van Doorn-Khosrovani et al., 2022) that specifically supports cost-efficiency studies (van Riel et al.). However, in an ideal setting, dose minimisation strategies should already be part of the initial drug development process.

## 3 Reducing waste

Reducing waste of medicines preserves valuable resources and minimises environmental impact. Dane et al. propose that same day scheduling of patients for the same treatment reduces waste, as prepared IV therapy for a no-show can be given to another patient. For the medicines administered by patients at home, which may have a high chance of being left unused (for instance, due to side effects, disease progression, or death), hospital pharmacies can deliver supply every 2 weeks or monthly to prevent spillage, though the environmental impact of more frequent deliveries should be carefully considered as part of a holistic strategy. Also collecting and re-dispensing unused oral anticancer drugs can reduce waste and save money (Dane et al.; Smale et al., 2023). However, to make this possible as part of routine care, local and regional regulatory guidance needs to be developed to determine the circumstances under which medicine re-dispensing is acceptable. In the absence of such guidance, oncologists should aim to prescribe just enough medication for patients to use at home, minimising the risk of wasting unused surplus when therapy changes are necessary (Dane et al.) while being mindful not to increase the burden of care for patients.

## 4 Combination therapies and challenges regarding their (cost)effectiveness

Assessment of a single technology is relatively straightforward, involving analysis of costs and outcomes for a particular intervention. However, assessing combination therapies or multiple technologies (several examples were submitted to our Research Topic) (Huang et al.; He et al.) is more complex. This complexity arises, for instance, from uncertainties regarding drug synergy, the cumulative financial burden and complex negotiations with various companies. Consequently, cost-effectiveness analysis of some new combination therapies show unfavourable high cost per QALY, even for high-income countries (Xiang et al.; Huang et al.).

In multiple myeloma, for instance, the current paradigm involves upfront triplet or quadruplet regimens, which can result in high toxicity and costs. It is unknown whether sequential use of these agents can lead to similar or even superior overall survival and quality of life. Walia et al. advocate for more trial data justifying the use of such multi-drug regimens.

In our Research Topic, He et al. discuss the challenges of economic analyses of combined technologies in first-line treatment for advanced hepatocellular carcinoma in China, where economic development varies significantly among provinces. Also, in the EU, diverse economic conditions and healthcare systems lead to varying cost-effectiveness thresholds among members, resulting in disparities in healthcare access.

Academic hospitals (Dane et al.) and cooperative study groups (Walia et al.) are well-suited to champion research agendas focused on studying the sequential use of therapies rather than combinations for relevant therapy classes, thereby reducing the burden of toxicity and contributing to the sustainability and affordability of healthcare systems.

## 5 In-house development and production

Academic hospitals often possess specialised expertise and the agility to develop and/or produce medicines in response to unmet medical needs. For instance, several ATMPs in use today had their initial prototypes developed in academic hospitals (Dane et al.). A recent EMA (European Medicines Agency) pilot aims to help academics further develop ATMPs. Netherlands currently reimburses an in-house adoptive cell therapy with tumour-infiltrating lymphocytes (TILs) (Rohaan et al., 2022) for advanced melanoma and two non-profit radiopharmaceuticals, prepared by hospitals (Dane et al.).

Compounding pharmacies can play a crucial role in producing medicines that are scarce or have been discontinued by pharmaceutical companies, ensuring ongoing access for patients. In some cases, compounded medicine can also serve as a cost-effective alternative to the commercial counterpart (Dane et al.; Bouwhuis et al.). Overall, academia-driven drug development could be instrumental in guiding novel public-private partnerships towards more affordable therapies.

## 6 Repurposing precision medicine in oncology

Drug repurposing uses approved medicines for new indications, offering alternative treatments or addressing unmet medical needs. This approach significantly lowers R&D costs, since these drugs have already passed safety assessments and demonstrated clinical efficacy. In the Netherlands, the Drug Rediscovery Protocol (DRUP) (van der Velden et al., 2019; van Waalwijk van Doorn-Khosrovani et al., 2019) an adaptive platform trial, provides off-label access to targeted therapies and immune-checkpoint inhibitors based on molecular tumour profiles. It offers treatment to patients who have exhausted standard-of-care options and generates necessary evidence for reimbursement. Currently, eighteen European countries collaborate in the PRIME-ROSE consortium (Precision Cancer Medicine Repurposing System Using Pragmatic Clinical Trials) (Taskén et al., 2024) to create a collaborative DRUP-like platform and accelerate drug development for rare indications. Also other EU funded platforms REMEDI4ALL and REPO4EU aim to boost drug repurposing.

Nevertheless, relying solely on public funding for drug repurposing trials may be unrealistic. Creating a clear path to drug registration and developing transparent, cost-based-plus pricing models that appeal to private investors can further stimulate drug repurposing. In the Netherlands, the centre for Future Affordable and Sustainable Therapy development (FAST) (de Visser et al., 2024) explores this area to better align innovation and affordability in drug development.

## 7 Conclusion

In this Research Topic, ‘Towards continued and affordable accessibility of innovative drugs: Sustainable development and efficient use of medicines’, we present a snapshot of ideas, insights, and ongoing efforts aimed at ensuring the continued and affordable accessibility of innovative drugs, as well as promoting sustainable development and the efficient use of medicines. It is important to realise that there is often a substantial knowledge gap after the launch of new drugs. This gap should be systematically and independently addressed to optimise treatment regimens. A recent European initiative is the Cancer Medicines Forum (CMF) (Saesen et al., 2022), a platform established to identify treatment optimisation questions and priorities, and help to address evidence gaps. National and regional funds supporting such initiatives can play a crucial role in improving cost-efficiency and reducing overtreatment.
